# Genetic Architectures and Cell-of-Origin in Glioblastoma

**DOI:** 10.3389/fonc.2020.615400

**Published:** 2021-01-21

**Authors:** Hyun Jung Kim, Jung Won Park, Jeong Ho Lee

**Affiliations:** ^1^ Graduate School of Medical Science and Engineering, Korea Advanced Institute of Science and Technology (KAIST), Daejeon, South Korea; ^2^ SoVarGen, Inc., Daejeon, South Korea

**Keywords:** glioblastoma, somatic mutation, neural stem cells, subventricular zone, genetically engineered mouse model

## Abstract

An aggressive primary brain cancer, glioblastoma (GBM) is the most common cancer of the central nervous system in adults. However, an inability to identify its cell-of-origin has been a fundamental issue hindering further understanding of the nature and pathogenesis of GBM, as well as the development of novel therapeutic targets. Researchers have hypothesized that GBM arises from an accumulation of somatic mutations in neural stem cells (NSCs) and glial precursor cells that confer selective growth advantages, resulting in uncontrolled proliferation. In this review, we outline genomic perspectives on IDH-wildtype and IDH-mutant GBMs pathogenesis and the cell-of-origin harboring GBM driver mutations proposed by various GBM animal models. Additionally, we discuss the distinct neurodevelopmental programs observed in either IDH-wildtype or IDH-mutant GBMs. Further research into the cellular origin and lineage hierarchy of GBM will help with understanding the evolution of GBMs and with developing effective targets for treating GBM cancer cells.

## Introduction

Glioblastoma (GBM) is a common, but aggressive, primary brain cancer of the central nervous system in adults and is associated with poor prognosis due to its invasiveness and resistance to therapy. According to 2016 WHO classification of glioma, GBMs are divided into: 1) IDH-wildtype (about 90% of cases), 2) IDH-mutant (about 10% of cases), and 3) IDH not otherwise specified (1). Molecular genetic features have emerged as fundamental factors contributing to its prognosis, particularly isocitrate dehydrogenase (IDH) mutation, which is considered a favorable factor. Whereas patients with IDH-wildtype GBM show a low median rate of survival of 14 to 16 months, patients with IDH-mutant GBM exhibit prolonged survival (median survival up to 31 months) and slower progression (1, 2). Over the past two decades, extensive and comprehensive genetic analysis of GBM has improved our understanding of GBM pathogenesis, and researchers have hypothesized that GBM arises from an accumulation of somatic mutations (3, 4). However, redundant signaling pathways and intratumoral heterogeneity underlie treatment failure and tumor recurrence (5–7). Thus, identifying the cellular origin of GBM would help with further understanding of tumor initiation/propagation and effective targets of use in treating GBM cancer cells. Regarding the cellular origin of cancer, cell-of-origin refers to normal cells in which oncogenic mutations first occur and accumulate to initiate tumor formation, while cancer stem cells (CSCs) refers to a subset of proliferating cancer cells that sustain tumor growth (8). The CSCs in GBM have been well-reviewed in many other papers (9–11). In this mini review, we mainly focus on the cell-of-origin in GBM and discuss the recent genomic analyses of GBM and genetically engineered mouse models (GEMMs) investigating tumorigenesis of GBM.

## Genetic Alterations in Glioblastoma

**Figure 1:**
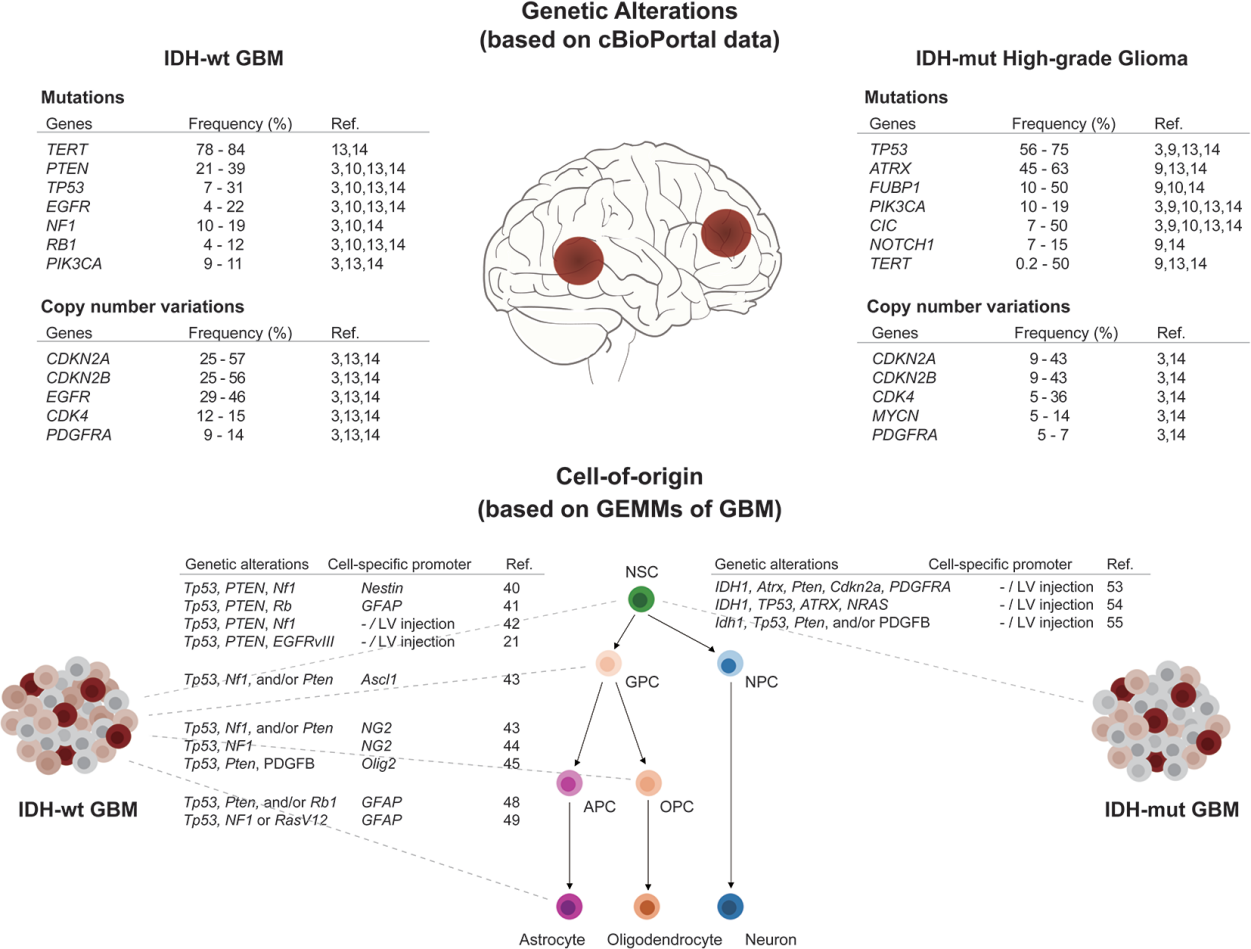
Overview of genetic alterations and cell-of-origin in IDH-wildtype and IDH-mutant GBMs. (Upper panel) Frequently occurring driver mutations and CNVs in IDH-wildtype and IDH-mutant high-grade gliomas (WHO grade 3 & 4) were listed as above. The frequencies were obtained from published data using cBioPortal (19, 20). (Lower panel) Multipotent neural stem cells (NSCs) have capability to self-renewal and differentiate into progenitors with restricted potential including glial precursor cells (GPCs), oligodendrocyte precursor cells (OPCs), astrocyte precursor cells (APCs), and neural progenitor cells (NPCs). Using specific genetic alterations and cell-specific promoters, NSCs and progenitor cells can be transformed to generate either IDH-wildtype or IDH-mutant GBMs in GEMMs.

Recent large-scale sequencing analyses have uncovered molecular alterations in somatic single nucleotide variants, copy number variations, gene expression profiles, and epigenetic signatures in GBM (3, 4, 12, 13). In addition, longitudinal genetic characterization of GBM has supported predictions of the order of mutation events and patterns of tumor evolution (14–18). Reviewing these studies, we summarize in the following paragraphs key somatic mutations, known as driver mutations, frequently occurring in IDH-wildtype and IDH-mutant GBM, respectively (**Figure 1**).

### IDH-Wildtype Glioblastoma

Although GBM is genetically and transcriptionally heterogeneous, previous studies have demonstrated concordant genetic alterations, including those in *TP53*, *PTEN*, *EGFR*, *PIK3CA*, and *PIK3R1*, *NF1*, and *RB1*, in human GBM samples (3, 4, 12). These mutations represent a set of deregulated signaling pathways, including growth factor (receptor tyrosine kinase [RTK]/phosphatidylinositide 3-kinase [PI3K]/Ras), p53, and retinoblastoma (Rb) signaling pathways. In the growth factor signaling pathway, *EGFR* is frequently activated with variant III deletion of the extracellular domain in GBM. Additionally, activating mutations in PI(3)K complex and inactivating mutations or deletions in tumor suppressor genes, such as *PTEN* and *NF1*, lead to uncontrolled proliferation. In the p53 pathway, inactivating mutations in *TP53*, along with *CDKN2A* (ARF) deletion, have been reported. Finally, deletions in *CDKN2A*/*CDKN3B* and amplifications of CDK4 have been found to result in Rb pathway inactivation, along with mutation or deletion of *RB1* itself. The majority of GBMs harbor genetic alterations in multiple signaling pathways, suggesting that these pathways are required for GBM pathogenesis.

Interestingly, up to 83% of IDH-wildtype GBMs exhibit telomerase reverse transcriptase promoter (*TERTp*) mutations (3, 21). The *TERTp* mutations, at positions 124 bp (C228T) and 146 bp (C250T) upstream of the *TERT* ATG site, generate *de novo* transcriptional factor binding sites leading to increased expression of *TERT* and subsequent telomere activation (21, 22). A recent study has demonstrated that IDH-wildtype GBM patients carry a high frequency of *TERTp* mutations in the astrocytic ribbon, the neurogenic niche of the postnatal human brain (23). This suggests that mutation of *TERTp* is an early shared event through which NSCs in the SVZ avoid replicative senescence, thereby increasing the possibility that these cells acquire GBM driver mutations (24). On the other hand, Körber and colleagues argued that *TERTp* mutations are subsequent mutations following copy number changes in *EGFR*, *PTEN*, or *CDKN2A* (14). These studies imply that among many GBM driver mutations, *TERTp*, *EGFR*, *PTEN*, or *CDKN2A* mutations seem to play a key role in the early stage of IDH-wildtype GBM formation.

### IDH-Mutant Glioblastoma

IDH-mutant GBM accounts for about 12% of all GBMs, with an occurrence rate of *IDH1/2* mutations of approximately 73% to 83% in secondary GBMs (12, 25). *IDH1/2* mutations have been observed in the vast majority of astrocytomas and oligodendrogliomas, and have been described as early molecular events during gliomagenesis (25, 26). Mutated *IDH1* elicits altered catalytic functions in metabolic, epigenetic, and reactive oxygen species managing pathways (27). GBMs with *IDH1* mutations show a higher frequency of loss-of-function mutations in *TP53* (3, 28). Based on a recent longitudinal study on IDH-mutant glioma, mutations in *IDH1* and/or *TP53* occur prior to *ATRX* alteration on the evolutionary trajectories of IDH-mutant gliomagenesis (24, 29). In addition, IDH-mutant GBMs exhibit alternating lengthening of telomeres (ALT) due to concurrent *ATRX* mutations, which are mutually exclusive with *TERTp* mutations (30, 31). Thus, genetic alteration enabling telomere maintenance are likely to be critical steps in GBM tumorigenesis.

Researchers have attempted to classify GBMs with similar molecular genetic characteristics into proneural, classical, and mesenchymal subtypes (32, 33). Each of these subtypes show an enrichment of lineage-specific gene signatures from distinct neural-glial lineages; for example, proneural GBMs show enrichment in oligodendrocyte precursor cell (OPC) genes (34). This implies that gene expression patterns in different subtypes may reflect the phenotype of their specific cell-of-origin.

## Cell-of-origin in Glioblastoma

To identify the cell-of-origin in GBM, understanding of normal cellular hierarchy is required. NSCs are ubiquitously found in all regions of the central nervous system during embryonic development and are capable of initiating cell lineages, leading to the formation of differentiated neurons and glial cells (35). NSCs give rises to intermediate progenitor cells with more restricted potential, which can proliferate and further differentiate into the three major cell types of the central nervous system. A subset of NSCs and lineage-restricted progenitor cells continue to reside in restricted regions of the postnatal and adult brain: the subventricular zone (SVZ) of the lateral ventricle and the subgranular zone (SGZ) of the dentate gyrus in the hippocampus (36, 37).

Considering that multiple oncogenic mutations are necessary for gliomagenesis, the self-renewal and proliferative properties of NSCs ensure appropriate conditions for endogenous accumulation of somatic mutations. Moreover, research has indicated that most driver mutations in cancer are attributable to DNA replicative errors, which are correlated with the total number of divisions of stem cells (38). Based on this notion, it has been hypothesized that the NSCs in the ventricular-subventricular zone is the main source of *de novo* somatic mutations throughout one’s lifetime. A recent study indeed showed that 55.5% of tumor-free SVZ tissue contains low-level mutations, such as *TP53*, *EGFR*, *RB1*, *PDGFR*, or *TERT* variations shared by matching tumor tissue in IDH-wildtype GBM patients, but not in IDH-mutant GBM patients (23). However, this study did not show any evidence of which cell type is the cell-of-origin in IDH-mutant GBM. Knowing now that human genetic studies provide the evidence of the cellular origin of IDH-wildtype GBM, we can recapitulate human GBM in mouse models, which are an invaluable tool with which to study the processes of tumorigenesis from originating cells (39–41). Below, we give an overview of GEMMs reflective of specific cell lineages and different combinations of GBM driver mutations, with or without IDH mutation (**Figure 1**).

### Animal Modeling of IDH-Wildtype Glioblastoma

To target NSCs in the adult brain, researchers utilized Cre recombinase-expressing adenovirus injected into the SVZ of mutant mice with conditional *Tp53*, *Pten*, and *Nf1* or *Rb* knockout, which resulted in the development of GBM (42, 43). Induction of the same tumor suppressor mutations in mice with *Nestin*-CreER transgenes also led to GBM formation (42). In addition, GBM has been successfully generated from NSCs harboring somatic mutations in *NF1*, *TP53*, and *PTEN* using *in utero* electroporation of CRISPR/Cas9 system (44). Similarly, *TP53* and *PTEN* mutations were introduced into the SVZ of conditional *EGFRvIII* transgenic mouse to generate a GBM (23).

Another model has suggested that GBM arises from committed precursor cells, such as glial precursor cells (GPCs), OPCs, and astrocytes. Researchers have used mice with an *Ascl1*-CreER transgene to target bipotential progenitors expressed in both adult neural and oligodendrocyte lineage progenitors (45). Bipotential progenitors carrying *NF1*, *TP53*, and/or *PTEN* mutations give rise to GBM, as do *NG2*-expressing OPCs (45–47). Several studies have suggested OPCs as the prominent cell-of-origin in GBMs, because of their aberrant growth prior to malignancy (23, 34, 47, 48). In contrast to glial lineage, susceptibility to malignant transformation declines with neural lineage restriction (49). Researchers utilized cell-specific promoters such as *Dlx1*, *Neurod1*, and *Camk2a* to introduce oncogenic mutations at specific time points during neural lineage specification.

There were several studies showing that mature astrocytes are also capable of tumor formation through de-differentiation. Loss-of-function mutations in *TP53*, *PTEN*, and/or *RB1* in GFAP-CreER mice (50) and injection of shNF1-shp53- or H-RasV12-shp53 lentivirus in the cortex of GFAP-cre resulted in glioma formation (51). The oncogenic virus induced astrocytes to de-differentiate into NS/PC-like state, by expressing the transcriptional factors Sox2, c-myc, and Nanog. The manipulation of pluripotency regulators are capable of inducing de-differentiation or cellular reprogramming (52, 53); however, the above studies have a limitation that GFAP-cre does not discriminate GFAP^+^ astrocytes from GFAP^+^ NSCs.

### Animal Modeling of IDH-Mutant Glioblastoma

The expression of *IDH1^R132H^* mutation in SVZ NSCs led to a proliferating phenotype, but it was insufficient to generate glioma (54, 55). Therefore, researchers have examined tumor-forming capacity by induction of additional oncogenic mutations in conjunction with *IDH1* mutation. Researchers utilized the RCAS-TVA system to express *IDH1^R132H^* and *PDGFA* in *Cdkn2a*, *Pten*, *Atrx* conditional knockout mice, thereby showing high-grade IDH-mutant glioma formation (55). Similarly, IDH-mutant glioma also was successfully generated by *IDH1^R132H^* and *NRAS* knock-in and sh*p53* and sh*ATRX* knockout in neonatal mice lateral ventricle using the Sleeping Beauty transposon system (56). Induction of *Idh1^R132H^* mutation with the loss of *p53* and *Pten* led to GBM formation using retrovirus expressing PDGFB-IRES-Cre recombinase and adenovirus expressing Cre recombinase (57). To date, all of IDH-mutant animal models mainly target NSCs in the SVZ; thus, additional animal studies need to be done to carefully examine the tumorigenic potential of other lineage-restricted cell populations following *IDH1^R132H^* and co-occurring oncogenic mutations.

Collectively, the cell-of-origin and subsequent mutant cell behavior appear to underlie different biological and genomic phenotypes in GBM. A recent study demonstrated that distinct characteristics in transcriptome profiles, obtained from GBM animal models targeting either NSCs or oligodendrocyte lineage cells, can be used to classify IDH-wildtype GBMs into two subtypes based on the cellular origin (58). However, individual cells from the same tumor harbor different mutations and exhibit diverse transcriptional patterns and phenotypes (59), making it difficult to completely unravel cellular origins and tumor evolution processes.

## Dissecting Cellular Hierarchy in Glioblastoma

With advances in single-cell sequencing, brain tumors have been examined at the single-cell level in an attempt to document developmental programs in GBM. Using single-cell whole-genome sequencing, researchers noted intratumoral clonal evolution based on *EGFR* aberrations (60). Patel and colleagues also showed the mosaic pattern of *EGFR* and other RTK signaling molecules (59). Despite the observed clonal heterogeneity in GBM, researchers have attempted to identify key neurodevelopmental programs from transcriptional profiles. Hierarchical clustering revealed that a subset of genes regulating oligodendrocyte function are important in primary GBM, along with genes related to the cell cycle, hypoxia, and complement/immune responses (59). Müller and colleagues also demonstrated that PDGF-driven GBMs exhibit a progressive induction of OPC-like cells (61). Additionally, several studies have recently indicated that IDH-wildtype GBM recapitulates a normal neurodevelopmental hierarchy (62, 63): malignant cells exist in four cellular states of distinct neural cell types, including NPC-like, OPC-like, astrocyte-like, and mesenchymal-like cells (62). Meanwhile, Couturier and colleagues demonstrated that putative originating cell populations share similar expression profiles of glial progenitors and that tumor cells are organized into the normal neural lineage hierarchy observed in fetal brain (63).

Although single-cell RNA sequencing of IDH-mutant GBM has not been conducted due to a small number of patients, several studies of IDH-mutant glioma have shed some light on the cellular hierarchy of IDH-mutant GBM. Therein, most malignant cells are differentiated into and are reminiscent of glial lineages (oligodendrocyte-like and astrocyte-like), while a small subset of cells remain undifferentiated, exhibiting features of NSCs (64, 65). Overall, aberrant differentiation toward glial lineage cells and developmental programs appears to dominate the cellular diversity in IDH-mutant glioma. These studies suggest that IDH-mutant GBM might originate from progenitor cells with more restricted potential.

## Discussion

A number of studies have described the cellular origin and hierarchy of IDH-wildtype GBMs in humans, and accumulating evidence from genome, transcriptome, and animal studies suggests that IDH-mutant GBMs have characteristics distinct from those in IDH-wildtype GBMs. This raises the hypothesis that IDH-mutant GBMs may arise from a different cell-of-origin that undergoes malignant transformation. Based on the hypothesis, we may consider another possible candidates for the cell-of-origin of brain tumor such as glial progenitor cells (66). Accordingly, additional genetic analysis and animal modeling of IDH-mutant GBM should be performed to identify the cell-of-origin. Furthermore, future research should seek to carefully characterize the underlying mechanisms of which cells initially acquire mutations and how mutation-harboring cells evolve and undergo lineage specification during gliomagenesis. Such research may benefit from focusing on influences from the tumor microenvironment (e.g., immune cell infiltration) on the fate of tumor initiating cells and subsequent expression-based subtypes in GBM.

## Author Contributions

HJK, JWP, and JHL conceived the topic for the mini review and wrote the manuscript. All authors contributed to the article and approved the submitted version.

## Funding

This study was supported by grants from the Suh Kyungbae Foundation (to JHL), from the Sovargen, Inc. (to JHL), from the National Research Foundation of Korea (NRF) funded by the Korea government, Ministry of Science and ICT (No. 2019R1A3B2066619 to JHL), and from the Daewoong Foundation (to HJK).

## Conflict of Interest

JHL is a co-founder and CTO of SoVarGen, which seeks to develop new diagnostics and therapeutics for intractable brain disorders.

The remaining authors declare that the research was conducted in the absence of any commercial or financial relationships that could be construed as a potential conflict of interest.

## Abbreviations

GBM, Glioblastoma; NSCs, Neural stem cells; IDH, Isocitrate dehydrogenase; GEMM, genetically engineered mouse model; RTK, receptor tyrosine kinase; PI3K, Phosphatidylinositide 3-kinase; TERTp, telomerase reverse transcriptase promoter; SVZ, Subventricular zone; ALT, alternating lengthening of telomeres; OPC, Oligodendrocyte precursor cell; SGZ, subgranular zone; GPC, glial precursor cell; APC, astrocyte precursor cell; NPC, neural progenitor cell.
